# Telephone-based reminder to improve safety after percutaneous coronary intervention

**DOI:** 10.1038/s41598-022-12722-3

**Published:** 2022-05-21

**Authors:** Jeffrey Chidester, Daniel Bennett, Chris Mathew, Tiffany Denkins, Rebecca Vigen, Tayo Addo, Sandeep Das

**Affiliations:** 1 University of Texas Southwestern, 6939 Patricia Avenue, Dallas, TX 75223 USA; 2 Revere Health, Provo, USA; 3 Parkland Health and Hospital System, Dallas, USA

**Keywords:** Interventional cardiology, Drug delivery

## Abstract

Dual antiplatelet therapy (DAPT) is a class I guideline indication after percutaneous coronary intervention (PCI). Our population is high-risk for low medication adherence. With a multidisciplinary team we developed a telephone-based intervention to improve DAPT adherence post-PCI. Patients undergoing PCI at our center were contacted by nursing staff via telephone at 1 week, 30 days, and 60 days post-procedure. Calls included a reminder of the importance of DAPT and elicited any patient concerns. Concerns were relayed to the team who could take appropriate action. For patients filling their medications at any pharmacies within our closed system the proportion of days covered (PDC) was calculated. These were compared to data for patients undergoing PCI in the seven months prior to program initiation. Information on interventions performed as a result of calls was also collected. During the study period, 452 patients underwent PCI. Of these, 70% were contacted and 244 filled their prescription at our system pharmacies. Twelve-month median PDC was 74%, with 45% of patients having PDC > 80%. There was no significant difference when compared to the group prior to the intervention, median PDC 79% and 50% of patients having PDC > 80%. In 26 patients calls led to interventions, removing barriers that would have otherwise prevented continued adherence. A telephone-based reminder system led to directed interventions in nearly 1 in 10 patients contacted. It was not able to significantly improve PDC when compared to a contemporary sample. This highlights the difficulty in using PDC to detect barriers to adherence.

## Introduction

Dual Antiplatelet Therapy (DAPT) to reduce in-stent thrombosis is a class I guideline indication after Percutaneous Coronary Intervention (PCI)^1^. Unfortunately, studies from our group and others have shown adherence to DAPT post-PCI among the urban poor to be suboptimal^2,3^. Follow up contact with adherence counseling, medication reconciliation and collaborative care has been shown to improve adherence^4,5^. With a team from Internal Medicine, Cardiology, Nursing, and Pharmacy, we developed a telephone-based intervention to improve DAPT adherence post-PCI.

## Methods

Patients who underwent PCI at our center from September 2017 through August 2018 were contacted by nursing staff via telephone at 1 week, 30 days, and 60 days post-procedure. We included all patients undergoing PCI and no pregnant patients or patients under the age of 18 were treated during the study period. Phone calls were made by staff fluent in English or Spanish depending on the documented preference of the patient, with a translation service utilized for other languages**.** A total of three calls were made to each patient at the specified time points. There was no specific script for the phone calls; however, calls included a reminder of the importance of DAPT and elicited any patient concerns surrounding their medications. These concerns were then relayed to the remainder of the team who were able to appropriately intervene. Interventions included scheduling appointments with a Physician or Pharmacist, altering of prescribed medication or filling pharmacy, or simply checking prescription status to provide patient reassurance. These interventions were documented.

For patients filling their medications at any of the 11 hospital-managed pharmacies within our closed system, the proportion of days covered at one year after PCI (PDC) was calculated based on manual chart review of pharmacy fill data. The formula for this value was simple, the number of days within the first year where patients were deemed to have had access to a prescription divided by 365 (PDC = [days with med available]/365). Adjustments were not made for recurrent admissions. Extra prescriptions being filled during a period in which medications were available by fill data were rare; however, when they occurred medication availability was assessed from this new date. These were compared to fill data for patients undergoing PCI in the seven months prior to program initiation, the only period during which this data was available prior to our project implementation. We used chi-square testing to compare the median PDC and proportion of patients with PDC > 80% in these two periods. Pharmacy data with specific fill dates and amounts for patients filling at hospital system pharmacies was available in both the pre-intervention and post-intervention groups. Due to the indigent nature of our population and the fact that prescriptions filled within the system are provided at zero or nominal cost, this constituted the majority of patients.

The University of Texas Southwestern Medical Center Institutional Review Board deemed this project non-regulated research and it was approved by the Parkland Health and Hospital System Research Office; accordingly, the requirement for informed consent was waived by both entities. As a result, patients did not have the intervention discussed with them and we did not require their consent prior to including them to participate. All methods were performed in accordance with relevant guidelines and regulations.

### Prior presentations

American Heart Association Quality of Care and Outcomes Research 2019 Scientific Sessions. April 5, 2019, Washington, DC.

High Value Practice Academic Alliance 2019 Conference. November 17, 2019, Baltimore, MD.

## Results

From September 2017 to September 2018, 452 patients underwent PCI. Of those patients undergoing PCI, 70% were successfully contacted by at least one phone call answered by the patient or a member of their household. One-third of patients were contacted on all three calls. Of all 316 PCI patients successfully contacted, 244 filled their DAPT prescription at our system pharmacies. Demographics for this group compared with patients in the seven months preceding the intervention are included as Table 1. In the intervention group there was a significantly higher proportion of patients with prior PCI. For this group, the twelve-month median PDC was 74%, with 45% of patients having PDC > 80%. Among the 316 patients contacted during our intervention, 26 required an intervention (Table 2). There was no significant difference in median PDC or percentage of patients with PDC > 80% when compared to the group prior to the intervention, with median PDC 79% and 50% of patients having PDC > 80% (Table 3). Characteristics of patients with low adherence and high adherence are presented in Table 4. Five patients were identified as deceased at the time contact was made and it is likely that additional patients who were not successfully contacted died during the study period.

**Table 1:** Demographics of patients.

	Pre-intervention	Intervention
Total	154	244
Age median (IQR)	58 (13)	58 (10)
Women n (%)	37 (24)	60 (25)
**Language**		
Spanish n (%)	65 (42)	87 (36)
English n (%)	82 (53)	143 (59)
Other n (%)	7 (5)	14 (6)
**P2Y12i**		
Clopidogrel n (%)	98 (64)	174 (71)
Ticagrelor n (%)	49 (32)	64 (26)
Prasugrel n (%)	7 (4)	6 (3)
**Comorbidities**		
Diabetes n (%)	82	143
Tobacco use n (%)	34	59
Prior CAD n (%)	56	105
Prior PCI n (%) *	46	99
History CHF n (%)	38	63
Depressed EF n (%)	27	37

*p < 0.05.

**Table 2:** Selected performed interventions.

Patient	Contact interval	Barrier	Response
Woman—58	30 days	No remaining medication	Clinic contacted, provided refill
Man—57	60 days	Unable to afford prasugrel	Prescription changed to clopidogrel
Man—59	7 days	Neglected to fill medication	Patient initiated fill
Man—47	30 days	Neglected to submit coverage paperwork	Given GoodRx coupon
Man—31	30 days	Coverage paperwork not yet processed	Low-cost refill ordered
Woman—50	60 days	Unable to afford medication for one week	Coordinated loading and maintenance dose at alternate pharmacy
Spouse of 'Woman—50'	n/a	Lack of medication	Provided alternate
Woman—60	7 days	Reported dizziness and confusion regarding medications	Appointment scheduled with pharmacist for education—she was taking all Metoprolol on Mondays and Lisinopril on Fridays

**Table 3:** PDC rates.

	Pre-intervention	Intervention
Mean PDC ± SD	69 ± 30	68 ± 29
Median PDC (IQR)	79	74
PDC > 80%, n (%)	66 (50)	109 (45)

**Table 4:** Demographics stratified by PDC above or below 80%.

	Pre-intervention		Intervention	
	PDC < 80%	PDC > 80%	PDC < 80%	PDC > 80%
Total	53	101	135	109
Age median (IQR)	56 (14)	59 (11)	57 (10)	61 (11)
Women n (%)	11 (21)	26 (26)	27 (20)	33 (30)
**Language**				
Spanish n (%)	24 (45)	41 (41)	45 (33)	42 (39)
English n (%)	27 (51)	55 (55)	83 (61)	60 (55)
Other n (%)	2 (4)	5 (5)	7 (5)	7 (6)
**P2Y12i**				
Clopidogrel n (%)	32 (60)	66 (66)	94 (70)	80 (73)
Ticagrelor n (%)	18 (34)	31 (31)	38 (28)	26 (24)
Prasugrel n (%)	3 (6)	4 (4)	3 (2)	3 (3)
**Comorbidities**				
Diabetes n (%)	26 (49)	56 (56)	77 (57)	66 (61)
Tobacco use n (%)	9 (17)	25 (25)	41 (30)	18 (16)
Prior CAD n (%)	20 (38)	36 (36)	56 (41)	49 (45)
Prior PCI n (%)	17 (32)	29 (29)	54 (40)	45 (41)
History CHF n (%)	16 (30)	22 (22)	35 (26)	28 (26)
Depressed EF n (%)	12 (23)	15 (15)	24 (18)	13 (12)

### Limitations

Our study investigated the implementation of a nursing-driven, multidisciplinary, patient-centered intervention aiming to improve the quality of care our patients receive. The characteristics of our patient population and health system as well as the design of our project as a quality improvement initiative impact the generalizability of our findings. Our patient population is a safety-net population with overall low socioeconomic status. Qualifying county citizens can receive medical care at low to no cost through a county assistance program, which gave us granular detail on their medication fills but also greatly reduces the barrier of out of cost expenses that other patients may encounter. However, these patients also have features such as unstable housing which can make consistent contact difficult. We restricted our analysis to patients who could be successfully contacted which may bias our findings towards patients more likely to have better medication adherence at baseline. As a result, our study indicates more about the impact of post-procedural contact in our patient population than it does about a system relying on telephone-based reminders in general.

## Discussion

This project was designed as a quality improvement initiative and much of its value came from analysis and adjustment of its implementation. A telephone-based reminder program identified adherence barriers in 1 of 10 contacted patients despite no detectable difference in PDC. This discordance is important as PDC is often used as a proxy for a patient actually taking a medication as prescribed. PDC can be readily calculated and implemented within EHR-based tools; however, factors including large volume fills, intercurrent hospital admissions, and overlapping prescriptions limit its ability to detect gaps in medication adherence. Furthermore, discontinuations or other changes would not be well captured. By contrast, direct patient contact enabled identification of barriers to adherence which would otherwise be clinically meaningful. This was perhaps the most important finding of the program; we had expected PDC to track well with actual adherence. We found contact success rate satisfactory; nevertheless, there was room for improvement in confirming patient contact information. The ability for rapid dissemination to a diverse care team enabled easy implementation of solutions to identified barriers. Finally, the barriers we identified through the calls were broad, including financial, medication side effects, and health literacy limitations. The personalized nature of the intervention allowed tailoring to these diverse needs. This low-resource project easily enabled us to contact and meaningfully impact a substantial proportion of our patients who have low health literacy and can be difficult to reach post-procedurally. In an era where telemedicine has become increasingly important, models that can improve patient’s care have characteristics that can benefit patients in other domains. In our case these included the use of a multidisciplinary team and patient-focused communication. Given the proportion of patients positively impacted, the practical superiority to EHR-derived PDC, and the positive response of involved team members, we plan to continue this program for all patients post-PCI at our institution to optimize their outcomes.
